# Fossils and plant evolution: structural fingerprints and modularity in the evo-devo paradigm

**DOI:** 10.1186/s13227-022-00192-7

**Published:** 2022-03-02

**Authors:** Alexandru M. F. Tomescu, Gar W. Rothwell

**Affiliations:** 1 Department of Biological Sciences, California Polytechnic State University Humboldt, Arcata, CA 95521 USA; 2 Department of Environmental and Plant Biology, Ohio University, Athens, OH 45701 USA; 3 Department of Botany and Plant Pathology, Oregon State University, Corvallis, OR 97331 USA

**Keywords:** Developmental regulation, Evo-devo, Fossil, Leaf, Modularity, Morphology, Rooting organ, Secondary growth, Strobilus, Structural fingerprint

## Abstract

Fossils constitute the principal repository of data that allow for independent tests of hypotheses of biological evolution derived from observations of the extant biota. Traditionally, transformational series of structure, consisting of sequences of fossils of the same lineage through time, have been employed to reconstruct and interpret morphological evolution. More recently, a move toward an updated paradigm was fueled by the deliberate integration of developmental thinking in the inclusion of fossils in reconstruction of morphological evolution. The vehicle for this is provided by structural fingerprints—recognizable morphological and anatomical structures generated by (and reflective of) the deployment of specific genes and regulatory pathways during development. Furthermore, because the regulation of plant development is both modular and hierarchical in nature, combining structural fingerprints recognized in the fossil record with our understanding of the developmental regulation of those structures produces a powerful tool for understanding plant evolution. This is particularly true when the systematic distribution of specific developmental regulatory mechanisms and modules is viewed within an evolutionary (paleo-evo-devo) framework. Here, we discuss several advances in understanding the processes and patterns of evolution, achieved by tracking structural fingerprints with their underlying regulatory modules across lineages, living and fossil: the role of polar auxin regulation in the cellular patterning of secondary xylem and the parallel evolution of arborescence in lycophytes and seed plants; the morphology and life history of early polysporangiophytes and tracheophytes; the role of modularity in the parallel evolution of leaves in euphyllophytes; leaf meristematic activity and the parallel evolution of venation patterns among euphyllophytes; mosaic deployment of regulatory modules and the diverse modes of secondary growth of euphyllophytes; modularity and hierarchy in developmental regulation and the evolution of equisetalean reproductive morphology. More generally, inclusion of plant fossils in the evo-devo paradigm has informed discussions on the evolution of growth patterns and growth responses, sporophyte body plans and their homology, sequences of character evolution, and the evolution of reproductive systems.

## Fossils provide invaluable evidence of evolution

Since their earliest occurrences in the fossil record more than 400 million years ago, vascular plants have diversified tremendously. While living species are characterized by a sporophyte that is differentiated into a wide array of organs and parts, including stems, leaves, roots, sporangia, seeds, cones, flowers, and fruits, the most ancient vascular plant sporophytes consisted of simple branching axes with terminal sporangia, a morphology that currently seems to have preceded the evolution of typical xylem and phloem [1]. Deeper in evolutionary time and plant phylogeny, bryophyte-grade embryophytes possessed sporophytes consisting of little more than a single sporangium [2]. The origin of the ancestral tracheophyte body plan and its transition from this simple organization to the complex sporophytes present in most modern tracheophyte lineages were accompanied by numerous dramatic changes in plant structure [3, 4]. Understanding these changes within a developmental framework is key to reconstructing plant evolution and phylogeny, and necessarily requires integration of data on developmental regulation, obtained from living plants, with data from the fossil record. To answer questions on the evolution of development, studies of living plants focus on careful studies of gene expression and function with the aim to gradually document regulatory pathways responsible for specific developmental processes. These studies have made significant strides toward understanding the principles of plant developmental regulation and have revealed the complexity of regulatory interactions which, even in model species, are often still largely unknown. Additionally, sequencing of algal streptophyte and plant genomes and transcriptomes over the last decade has opened opportunities for predicting the makeup of gene regulatory networks in different lineages, thus informing hypotheses about the evolution of these networks (e.g., [5, 6]).

Understanding morphological evolution—i.e., evolutionary changes in plant structure—within a developmental framework also necessitates in-depth knowledge of both the intermediate stages that populate the different trajectories of evolutionary change, and of the order in which they occurred within each lineage. Most of these intermediate stages of evolutionary changes that have transformed plants over time are not present among species of the modern flora, and are preserved only in the fossil record. Therefore, fossils provide vital evidence, unavailable otherwise, for understanding the origins of modern plant structure and for reconstructing the *patterns* of structural changes through time that have produced the morphological diversity that characterizes modern vegetation.

## At macroevolutionary scales, ecological crisis is the driver of evolution

Of equal importance for documenting structural change through time, the fossil record provides convincing evidence for the fundamental *processes* that underlie plant evolution [3]. While evolution traditionally has been explained within the context of classical and population genetics, as early as the 1970s tests of those traditional evolutionary hypotheses using paleontological data began to reveal patterns of change over broader temporal scales additional to those predicted by genetics-based evolutionary theory at the population level [7]. Population genetics theory predicts that natural selection is the driving force for evolutionary change and that such selective forces are most impactful within well-established ecosystems. At macroevolutionary scales, the paleontological record reveals that the most rapid evolutionary diversifications occur immediately after biological catastrophes, at moments when extinction has dramatically reduced biotic selective pressures and has opened up vast swaths of ecological space for colonization by new species [8, 9]. Both macroevolutionary theory (e.g., [10]) and paleontologically established patterns of evolutionary change indicate that the reestablishment of complex plant communities leads to long periods of evolutionary stasis that witness only small-scale selection-driven evolutionary modification [11, 12]. Therefore, the fossil record reveals that evolutionary diversification is the least rapid when genetically based evolutionary theory predicts it should reach its highest rates. This apparent contradiction is mostly a matter of scale—natural selection acts primarily as a filter removing maladapted phenotypes at microevolutionary scales, and less as a driving force at macroevolutionary scales [13–17]—and this is especially apparent within a paleontologically sanctioned context.

## The evo-devo paradigm and the role of morphology

A growing appreciation for the role of development in evolution, whose understanding has been dramatically elevated by the advent of developmental molecular biology, has fostered an improved perception of evolutionary process [18, 19]. Within this context, we now recognize that the genome encodes the program that determines ontogeny, and it is that program which evolves through time [3]. For each organism, the genetic program is implemented through the processes of development. As a result, the phenotype of an organism at any point during its ontogeny represents the cumulative structural evidence for the developmental processes that generated it. In turn, these reflect the deployment of the genetic program, i.e., the activity of regulatory mechanisms that direct those developmental processes. Therefore, changes in the genetic program produce predictable changes in the phenotype of the resulting organisms, which we refer to as morphological evolution [20].

**Fig. 1:**
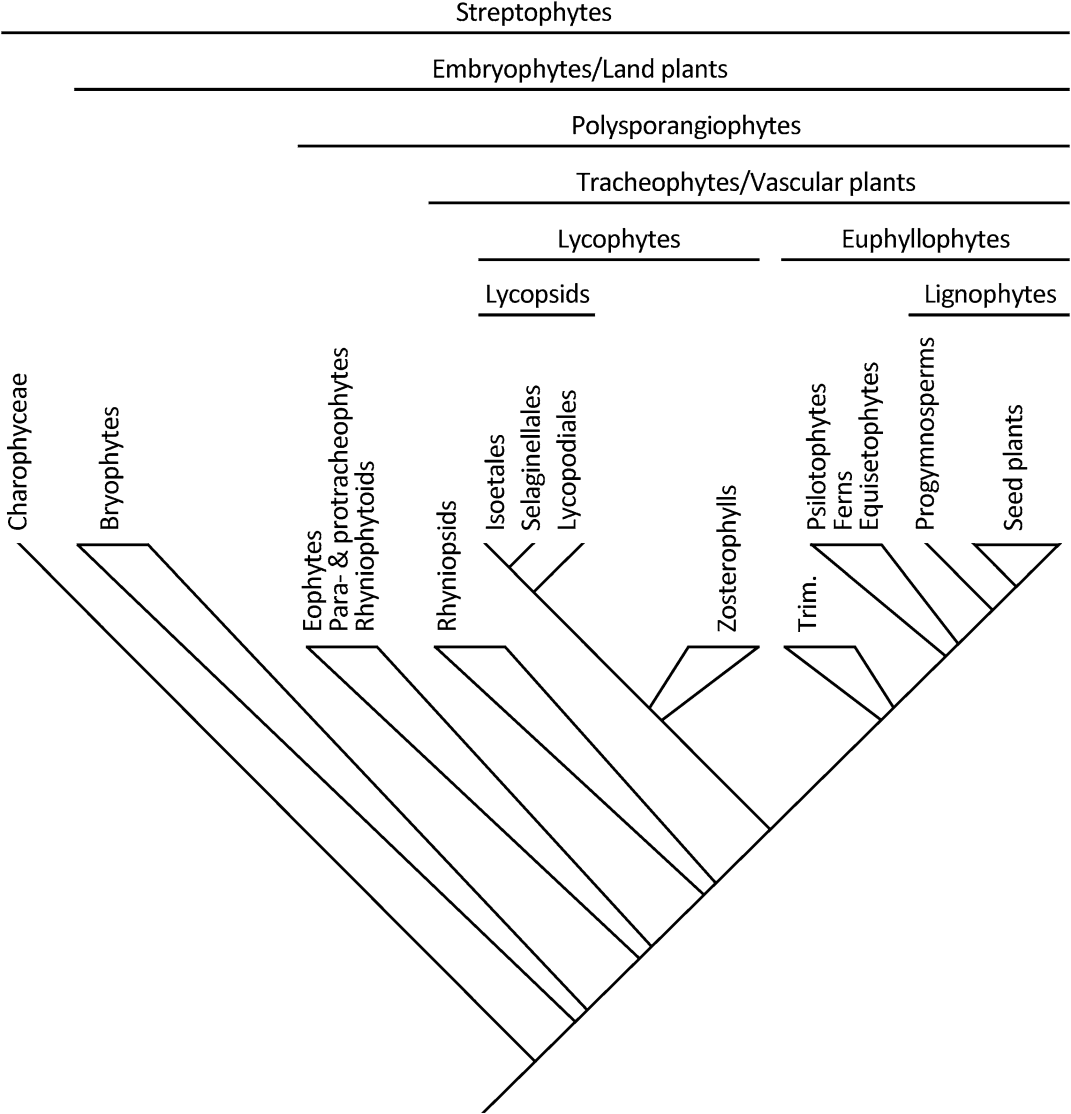
A phylogenetic framework for the groups discussed throughout the paper; a polygon denotes uncertainty in the relationships among different lineages of that group (mostly due to conflict between the results of different analyses); Trim = Trimerophytes

For example, the origin of branching in the sporophyte phase of the embryophyte life cycle was, alongside the evolution of xylem and phloem, a seminal event leading to the evolution of vascular plants [21]. That change may be hypothesized to have resulted from the prolongation of the time during which the sporophyte underwent apical growth before going through a transition to reproductive growth and the production of terminal sporangia [3, 4]. According to this hypothesis, a change in developmental regulation in the early sporophyte stage of bryophyte-grade plants resulted in the origin of potentially indeterminate growth from an apical meristem which, in turn, allowed for branching. Evidence in support of this hypothesis is provided by apogamous sporophytes of the living moss *Physcomitrium patens*, wherein the combination of gene silencing and auxin transport inhibition produces a comparable heterochronic change that results in an elongated and branched axial sporophyte body [22]. Indeed, the oldest known branched sporophytes, which characterize the polysporangiophyte clade (Fig. 1), show bryophyte-grade features, such as bryophyte-type photosynthate-conducting cells in the absence of true tracheids [1] and nutritional dependence on the gametophyte phase [23, 24].

**Fig. 2:**
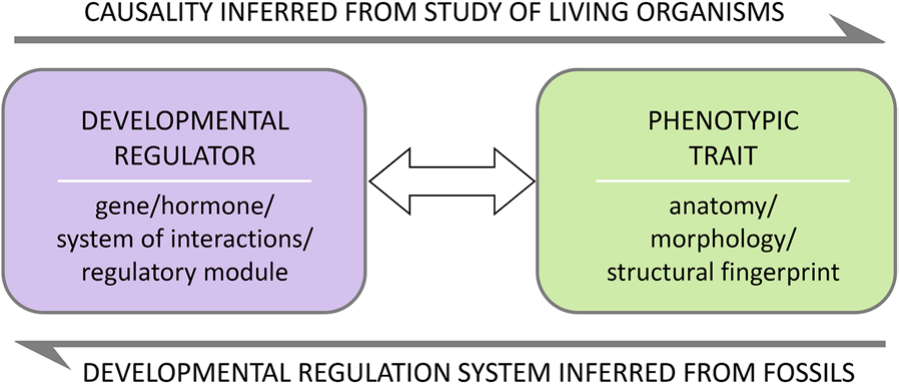
Anatomical and morphological features seen in organisms bear witness to the activity of specific regulatory modules. Studies of living organisms can identify the regulatory entities of specific developmental processes, which produce well-defined phenotypic traits. Such phenotypic traits, thus, represent structural fingerprints of the deployment of those developmental regulators. In turn, identification of structural fingerprints in fossils provides evidence for the activity of their corresponding regulatory entities in extinct lineages, informing the evolutionary history of those regulators

Because phenotypes are the direct result of development under the control of genetic regulation, the wedding of paleontology (i.e., phenotypes through time) with regulatory genetics (i.e., genomic changes leading to phenotypic changes) provides a framework for understanding the evolution of development (Fig. 2). Within this context, the developmental underpinnings of morphology take on a much more central role in understanding both the patterns and processes of plant evolution, and the fossil record provides access to direct evidence of that evolution. Building on data and ideas published by ourselves and others, here we focus on the modular nature of developmental regulation emphasizing the role of fossils in supporting or generating hypotheses on modularity and its role in morphological diversity and evolution. These have never been considered together in a comprehensive discussion of the role of fossils in documenting the modular nature of development and its regulation, which have otherwise been widely discussed in “neontological” evo-devo.

## Developmental regulation is modular and hierarchic

Throughout the ontogeny of an organism, developmental regulation is a complex, dynamic system of physical interactions between proteins, hormones, small RNAs, etc. An important feature of this system is that the strength and duration of interactions between its different components change during ontogeny; the changes separate subsets of strongly interdependent interactions that can be regarded as distinct regulatory modules (e.g., the variational modules of Pavlicev and Wagner [25]). Thus, the modules are subsets of the broader system of interactions; they are tightly integrated internally, on one hand (i.e., within-module interactions are strictly dependent on each other), and on the other hand are independent from, or more loosely integrated with, other such subsets of interactions (i.e., modules can be turned on and off without affecting the activity of other modules) [26]. Additionally, interactions among regulatory modules can be hierarchical.

In an experimental study of vascular cambial growth in the angiosperm *Ficus*, Lev-Yadun [27] demonstrated that girdling induces transient production of wood in which rays develop normally but the axial system exhibits dramatically altered anatomy, differentiating into isodiametric parenchyma. Although the study did not address directly the specific genetic and molecular factors that control these developmental processes, this example illustrates both modularity and hierarchy of modules in developmental regulation. The different effects that the girdling treatment had on the anatomy of the two systems of secondary xylem—radial (rays) and axial (tracheary elements, xylem parenchyma, fibers)—indicates that the two aspects of development can proceed independently of each other. In turn, this implies that their regulation is uncoupled and, thus, distinct regulatory modules, at least in terms of patterns of cell division and differentiation. Girdling, on the other hand, did not directly affect the three-dimensional organization of secondary xylem (into two distinct systems), which suggests that this organization is controlled at a different hierarchical level of developmental regulation.

Evidence for modularity in developmental regulation abounds in all biological systems and discussions of developmental modularity provide a meeting place for developmental and evolutionary biologists [28]. The evidence is often provided by regulatory mechanisms whose activation—or lack thereof—is independent of their broader regulatory context, ontogenetic timing, or position of deployment, thus indicating that they represent distinct regulatory modules. Such are the several regulators that induce different histological differentiation or morphogenetic effects which, in different combinations, are responsible for distinct morphologies that bridge the reptilian scale to avian feather spectrum of tetrapod skin appendages [29]. In plants, we see examples of modularity when different aspects of the development of the same tissue, tissue system or organ are controlled independently: the control of vascular proliferation and vascular organization that are genetically separable [30]; xylem and phloem cell differentiation from procambium controlled independently of the neat separation of the two tissues within vascular bundles [31]; differentiation of secondary phloem and secondary xylem controlled independently of each other [32]; blade expansion and leaflet initiation uncoupled during compound leaf morphogenesis [33]; floral organ length and corolla limb dimensions varying independently in two closely related species of the same genus [34].

Another example is the reiteration of structural modules consisting of four nuclei (whose makeup is likely determined by the same regulatory mechanism) among the diverse types of angiosperm megagametophyte development [35]. Along similar lines, the same set of regulatory interactions may be deployed in different locations within the plant, like in the case of a module that regulates cell wall remodeling, expressed in both lateral root emergence and petal abscission [36]; and, more generally, in the development of ectopic structures of many kinds. Conversely, different developmental fates can be determined in cells that share the same identity by the action of distinct regulatory modules, such as pericycle cells induced into either lateral root primordium founders or cork cambium initials by the integration of different developmental cues into distinct regulatory modules [37]. At a broader biological scale, there is evidence for regulatory mechanisms transferred between the gametophyte and sporophyte generations [38–42].

In an evolutionary perspective, the modularity of developmental regulation allows for broad variation in the organization of ontogenetic trajectories over evolutionary time, with different phenotypic outcomes in different organisms. The roots of variability reside in the degree of integration of the modules, which can be more or less tightly integrated—i.e., interacting with, influenced by or dependent on, each other—in ways that can be hierarchical or not. Variability also arises from the combinatorial nature of the activation (or lack thereof) of different modules—i.e., different modules being turned on or off separately or in concert—at different stages in ontogeny. Together, these sources of variation underpin a vast amount of potential diversity in ontogenetic trajectories, able to generate an equally vast amount of potential phenotypic diversity. Such potential provides the raw material for morphological evolution. Thus, for example, analyses of plant comparative morphology across evolutionary time and phylogenetic space have assembled data that indicate different pathways of accretion of complexity in different lineages [43, 44], and support hypotheses about the evolution of morphological complexity as a mosaic of features combined in different ways and assembled in different sequences in different major lineages [44, 45].

## Structural fingerprints provide evidence for the deployment of regulatory modules across phylogeny and time

**Fig. 3:**
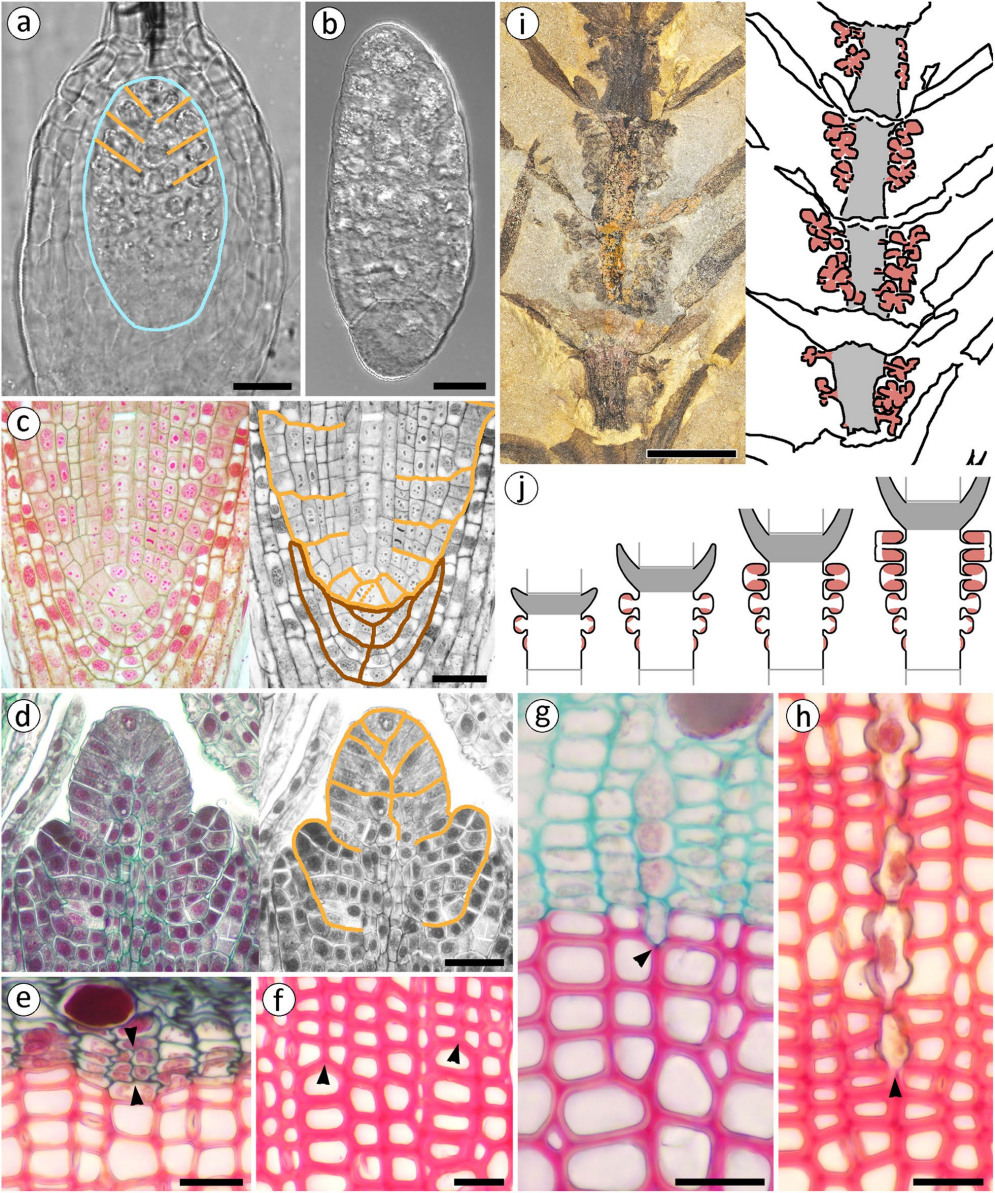
In plants, cells are attached to each other by their walls, in the position in which they arise by cell division. As a result, the relative arrangement of cells records sequences of cell division, allowing for reconstruction of developmental processes. The arrangement of cells at the tip of *Physcomitrium* moss embryos (**a**, **b**) reveals growth from an apical cell (images courtesy of C. Jill Harrison); embryo outlined in blue inside the archegonium in a, orange lines emphasize the cells arrangement. The patterning of merophytes formed from derivatives of the apical cell is easily distinguishable in longitudinal sections of *Equisetum* root (**c**) and shoot (**d**) apical meristems and reflects the sequence of divisions of the apical cell; root and stem merophytes traced in orange; root cap merophytes traced in brown in c. Anticlinal (multiplicative) divisions (between arrowheads in **e**) of vascular cambium initials produce additional files of cells observed in cross sections of secondary tissues (in a *Pinus* stem). “Doubled” tracheid files (arrowheads in **f**) are fingerprints that reveal the exact location and timing (measured in wood thickness or growth rings) of symmetric divisions of the cambial initials. Asymmetric divisions of cambial initials initiate rays (arrowhead in **g**), whose inner ends (arrowhead in **h**) mark the position and timing of the asymmetric division. Patterns of sporangiophore numbers and sizes along fertile internodes of the Permian equisetalean *Cruciaetheca* (**i**) record the basipetal direction of tissue and organ maturation within internodes (**j**), generated by growth from intercalary meristems; sporangiophores in red, in the image tracing in i and in the diagram in j; internodes gray in i; nodes gray in j; j modified from [47]. Scale bars 20 µm in (**a**, **b**); 50 µm in (**c**, **d**); 20 µm in (**e**–**h**); 1 cm in (**i**)

The anatomical and morphological features seen in organisms bear witness to the activity and, sometimes, interactions of specific regulatory mechanisms. When a specific developmental process can be matched with specific anatomical or morphological features, those features represent *structural fingerprints* of the activity of regulatory mechanisms that control that process (Fig. 2). In other words, in such cases studying morphology can teach us about developmental regulation. In plants, specifically, identification of such fingerprints is facilitated by the fact that the position of cells is largely fixed; cells are attached to each other by their walls, in the position in which they arise by cell division. As a result, the relative arrangement of cells records sequences of cell division, allowing for more detailed reconstruction of developmental processes. Such is, for instance, the easily distinguishable patterning of merophytes that form from immediate derivatives of the apical cell and show corresponding arrangements around and behind the latter in bryophyte or equisetalean apical meristems (Fig. 3a–d); or the arrangement of cells in cross sections of secondary tissues, which records the sequence of past periclinal and anticlinal divisions in the cambial initials (Fig. 3e–h). Similarly, at a larger scale, the patterns of sporangiophore (i.e., fertile appendage) numbers and sizes along equisetalean fertile internodes (Fig. 3i) record the polarity of meristematic activity in intercalary meristems (Fig. 3j).

Although genetic regulation, which results in structural features of plants, is a transitory process not available for direct examination from fossils, these features—structural fingerprints—do accurately reflect the regulatory genetics by which they were produced. Therefore, when structural fingerprints are identified in fossils, they can be employed to infer the specific regulatory mechanisms by which they developed [46] (Fig. 2). Because in living plants we can tie these fingerprints to specific, detailed regulatory mechanisms, we can circumscribe the exact nature of regulatory modules and of interactions within and between modules that generate the structural fingerprints. If compared between extant plant lineages, this type of information can reveal the degree of variation in the structure and interactions of regulatory modules that characterize different lineages. Studies that integrate structural fingerprints and molecular-genetic regulation in living plants allow us to infer the same relationships between gene regulation and structure in extinct plants. This opens up a whole new window onto the evolution of development, by allowing us to trace the presence of regulatory processes and the activity of specific regulatory modules in phylogenetic space and evolutionary time. In other words, this methodology allows us to connect the regulatory genetics of living forms to their long-extinct ancestors and precursors (or, at least, to form hypotheses about such connections) within an empirically based framework (e.g., [47]).

For example, if a particular regulatory pathway is shared by sister clades, then we can hypothesize that they share a common developmental tool kit which has been inherited from a common ancestor that possessed that tool kit [3]. Those hypotheses can then be tested by searching for the structural fingerprint of those tool kits in specimens of the common ancestor (or extinct sister group) of the two clades. An example of that sort of hypothesis test is presented below for the role of polar auxin regulation in the development of vascular tissue.

## A quintessential structural fingerprint and its implications

Regulation of both primary and secondary vascular tissue production (specification, differentiation) by the directional transport of auxin (polar auxin transport) is probably a common denominator of development in vascular plants, wherein it evolved increasing sophistication at successively more derived levels of their phylogeny [3, 48, 49]. Studies in angiosperms have demonstrated that polar auxin transport and the auxin gradients it generates, established early in embryogenesis, are responsible for primary vascular architecture (procambium specification, vascular tissue differentiation), as well as cambial identity and functioning in secondary growth (e.g., [50–54]). During secondary growth from a vascular cambium, polar auxin regulation of cell positioning and growth direction produces characteristic circular patterns in specific positions in the secondary xylem. These patterns consist of swirls of tracheary elements positioned upstream of locations where polar auxin flow in the cambium has been impeded by obstructions, such as axillary buds and lateral branches [48, 55, 56]. Such “auxin swirls” therefore represent anatomical fingerprints for polar auxin regulation of secondary xylem patterning and were the first structural fingerprint to be recognized in fossil plants [3, 46].

**Fig. 4:**
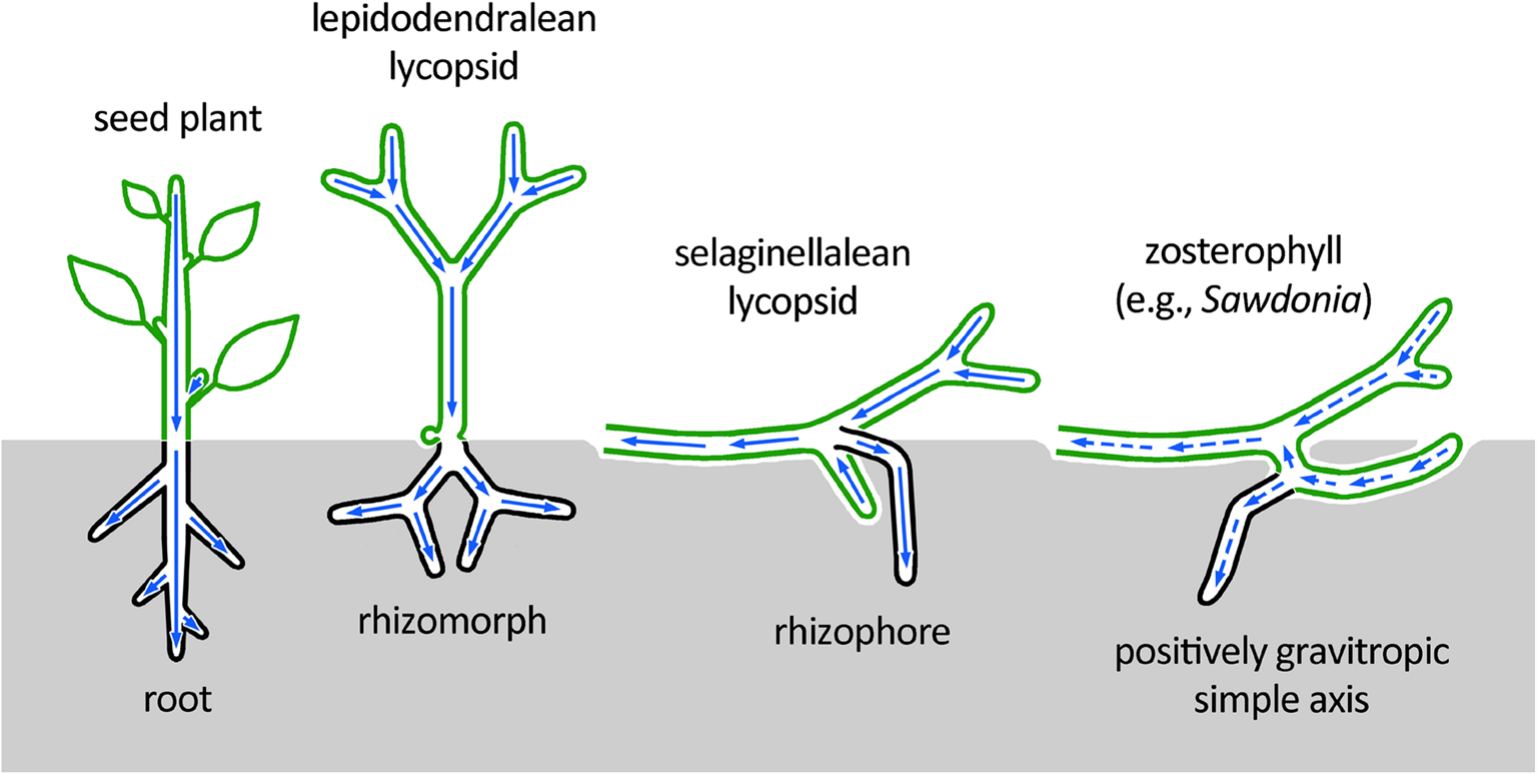
In contrast to the shoots (green), polar auxin transport (PAT; depicted by blue arrows) is acropetal in the roots of seed plants and the rhizophores of selaginellalean lycopsids, whose homologies are equivocal. Additionally, fingerprints for the directionality of PAT demonstrate that the rhizomorphs of lepidodendralean lycopsids, which are shoot homologs with rooting function, also had acropetal PAT [60]. This shared directionality of PAT implies that acropetal auxin flow transcends organ identity and is more tightly linked to the positively gravitropic response or rooting function of axes (gray), independent of their homology. In turn, this suggests that the positively gravitropic axes with rooting functions produced by K-branching in zosterophylls with simple body plan may also have had acropetal PAT (dashed blue arrows)

Because xylem has excellent fossilization potential, wood anatomy is among the most common sources of data in the plant fossil record. Herein, swirls of tracheary elements provide powerful evidence (1) for polar auxin transport as a regulatory mechanism of tissue patterning during vascular cambial growth shared among several major plant clades [57], and (2) for the antiquity of polar auxin regulation in secondary tissue patterning. This demonstrated shared mechanism suggests that at least some of the basic regulatory elements in the control of secondary growth may have been part of a developmental toolkit shared among all euphyllophytes, or even all tracheophytes [45, 58]. The same structural fingerprint identified in the rooting structures (rhizomorphs) of Pennsylvanian (c. 310 million-years) arborescent lepidodendralean lycophytes demonstrated that these positively gravitropic axes have acropetal auxin transport, unlike the shoots to which they are homologous [59], and similar, instead, to other rooting structures with different homologies [60, 61]. In turn, this shared directionality of polar auxin transport implies that acropetal auxin flow transcends organ identity and is more tightly linked to positively gravitropic axes, independent of their homology [62]—whether they be roots (as in most extant tracheophytes), modified shoots (in isoetalean and lepidodendrid lycophyte rhizomorphs, and in drepanophycalean lycophyte rooting axes), rhizophores (in *Selaginella*), or simple undifferentiated axes (in zosterophylls) (Fig. 4).

## Combining structural fingerprints

By the beginning of the Silurian (444 million-years ago), the first members of the clade characterized by branched sporophytes and including all vascular plants (i.e., polysporangiophytes), had emerged [63] out of a plexus of early embryophytes whose earliest bryophyte-grade representatives go at least as far back as the Middle Ordovician (468 million-years ago; [64]). If trilete spores are, indeed, exclusively characteristic of vascular plants and not of all the embryophytes, as has been proposed by Steemans et al. [65], then vascular plants and, by extension, polysporangiophytes may have evolved as early as 455 million-years ago, around the beginning of the Late Ordovician [66, 67]. Direct information on these plants is available exclusively from fossils, which provide multiple structural fingerprints that when combined, allow us to reconstruct the morphology and life history of these tracheophyte ancestors.

Early polysporangiophytes had diminutive sporophytes that were only little more than branched versions of bryophyte-grade sporophytes [4]. The small size of the sporophytes is immediately apparent in the fossils [23, 63]. The branching of these sporophytes indicates that they grew from apical meristems, but their diminutive size, scant branching, and presence of sporangia terminating all branches (e.g., [1, 4, 63]) indicate that their meristematic growth was determinate. The small size of these sporophytes also supports the hypothesis that they were nutritionally dependent on the gametophytes [23], like the sporophytes of bryophytes. The dependence of sporophytes on the gametophytes is also consistent with the inference that their growth was determinate. Recently it has become apparent that these early sporophytes had specialized photosynthate-conducting cells similar to those of bryophytes [1], but it is less clear whether the earliest polysporangiophyte sporophytes possessed tracheid-based water-conducting tissues, as the oldest tracheids discovered to date are significantly younger—424 million-years old [68]. This is close to the (slightly older, ca. 432 million-years) age of the oldest known sporophytes that reached sizes consistent with physiological independence [69].

The gametophytes that supported these diminutive sporophytes were probably thalloid, like those of hornworts and liverworts and unlike those of younger, Devonian (c. 410 million-years old) polysporangiophytes such as *Aglaophyton*, *Rhynia*, *Horneophyton* or *Nothia* [70]. This inference is based on the thalloid form of fossils found associated with (but not attached to) branched sporophytes in Silurian and Early Devonian layers at multiple locations [71, 72]—some of which bear transfer cells typical of the gametophyte–sporophyte connection in bryophytes [24]—and on structural and chemical evidence that some of these fossils are plants [73–75].

## Echoes of modularity

### The leaves of ferns and seed plants

The Euphyllophytina is the largest and most diverse of the two major clades of living vascular plants, and is represented in the modern flora by seed plants (flowering plants, gymnosperms) and several lineages of seed-free plants (marattialean, ophioglossalean, and leptosporangiate ferns, equisetaleans, and psilotaleans—i.e., *Psilotum* and *Tmesipteris*). The overwhelming majority of the extant euphyllophytes show stem–leaf–root organography in their vegetative sporophyte; exceptions include a few highly derived angiosperms with reduced or incompletely differentiated sporophytes (e.g., Podostemaceae, Lemnaceae), ferns (e.g., *Salvinia* lacking roots) and psilotaleans (which lack roots entirely and whose lateral appendages may or may not be reduced leaves; [76]). Because of this, early phylogenetic analyses of living species have inferred that the derived stem–leaf–root organography has evolved only once among euphyllophytes [77]. However, all members of the basal grade of fossil euphyllophytes, referred to as trimerophytes, have plesiomorphic sporophyte morphology consisting of simple branching axes that were vascularized and bore sporangia, but were not differentiated into roots, stems and leaves. The absence of leaves in the trimerophytes, coupled with their phylogenetic position among euphyllophytes [21, 78], and with fossil evidence for the evolution of euphyllophyte leaves [79, 80], provide compelling evidence that leaves evolved independently and in parallel, from such leafless trimerophytes, in several different euphyllophyte lineages [81]. Thus, the leaves of different euphyllophyte clades that appear to be homologous to neontologists, actually resulted from parallel evolution [78–82]. This is one of the most compelling examples of fossils and morphology allowing for the recognition of analogy (or homoplasy; similar characters in two groups that evolved independently by parallel or convergent evolution) and its distinction from homology (i.e., characters in two groups that are inherited from a common ancestor that had those characters).

Two main structural changes that have led to the evolution of leaves from leafless trimerophyte axes are (1) the change from indeterminate to determinate growth, and (2) the origin of abaxial–adaxial patterning in the transition from radial to bilateral growth [83]. Leaves of living euphyllophytes typically have both determinate growth and bilateral (abaxial–adaxial) polarity, and available evidence suggests that each of these properties is controlled by distinct regulatory modules. Because data available currently on gene expression patterns offer only a spotty coverage of the taxonomic breadth of living euphyllophytes, and because those data are not matched in terms of taxonomic coverage by data on gene function, inferences on gene functions in different lineages can only be tentative at this point. Nevertheless, recurrent patterns of expression, some of which are complemented by functional data, provide indications on putative gene functions. For instance, meristematic activity at the shoot apex is probably maintained by *class I KNOX* genes and *LFY* in both ferns and angiosperms (at least insofar as this can be predicted based on studies in model species). These genes are probably also responsible for proliferative growth in the leaves of both ferns and angiosperms (e.g., compound leaves) [81, 84–87]. Thus, determinacy of growth in leaves may well reflect the evolution of regulatory mechanisms that repress these genes, such as the ARP group genes that repress *KNOX I* gene activity. Similarly, adaxial–abaxial polarity (sometimes referred to as dorsiventral polarity) seems to result from the expression of, and interactions between, class III HD-ZIP genes (promoters of adaxial identity) and *KANADI* genes (promoters of abaxial identity), in all euphyllophytes [88–90].

**Fig. 5:**
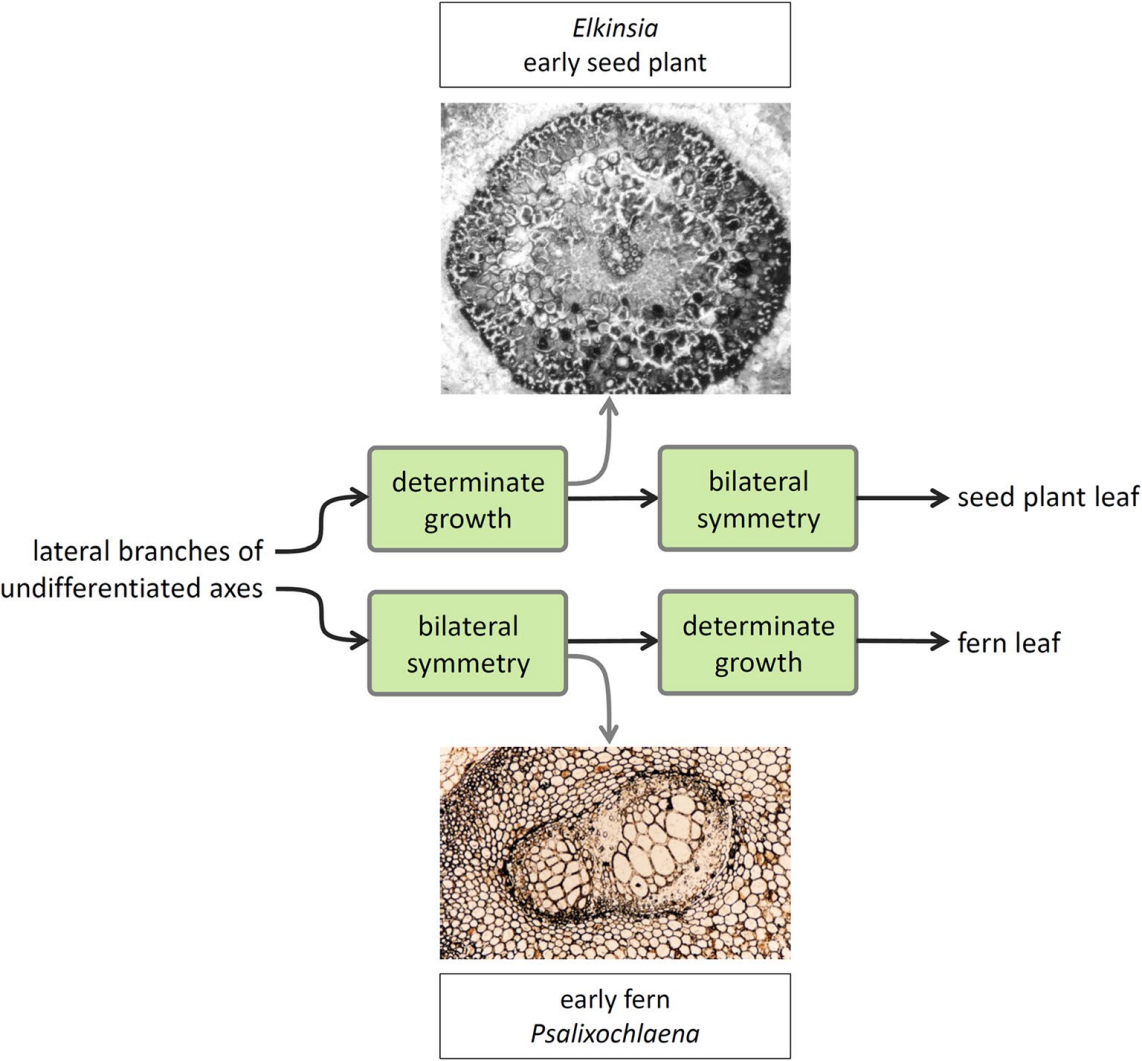
Euphyllophyte leaves are thought to have evolved from lateral branching systems like those seen in early representatives of the clade (e.g., *Psilophyton*). Structural fingerprints for adaxial–abaxial polarity (dorsiventral polarity) observed in fossils indicate that whereas seed plants evolved determinate growth before adaxial–abaxial polarity in the leaves, in filicalean fern leaves evolution of adaxial–abaxial polarity preceded determinacy. The early fern *Psalixochlaena* exhibits adaxial–abaxial polarity in its leaves (i.e., protoxylem on the adaxial side and phloem on the abaxial side of the leaf vascular bundle cross-sectioned in the figure), which had indeterminate growth; in contrast, the leaves of the early seed plant *Elkinsia* had determinate growth but their vascularization had radial symmetry (protoxylem surrounded by metaxylem in the vascular bundle cross-sectioned in the figure), at least in their terminal segments. This observation provides one of the lines of evidence supporting independent evolution of leaves in ferns and seed plants

Adaxial–abaxial polarity is reflected in the flattened morphology of leaves and, even in the absence of this morphology, can be ascertained based on the bilateral patterning of the vascular tissues that supply these lateral appendages (i.e., phloem positioned abaxially and xylem adaxially). Using structural fingerprints for the two leaf-defining features—leaf morphology for determinate growth and polarity of leaf vascular tissues for adaxial–abaxial polarity—and querying the fossil record of early ferns and seed plants, Sanders et al. [79] demonstrated that whereas seed plants evolved determinate growth before adaxial–abaxial polarity in the leaves, in filicalean fern leaves evolution of adaxial–abaxial polarity preceded determinacy (Fig. 5). Aside from supporting hypotheses of independent evolution of leaves in ferns and seed plants, reflecting different trajectories in terms of sequence of character evolution, this is consistent with a modular nature of the regulators of leaf determinacy and adaxial–abaxial polarity, which allows for independence in the deployment of these two features. Thus, structural fingerprints for developmental mechanisms preserved in fossils provide evidence for the modular nature of specific aspects of leaf developmental regulation.

Venation is an additional facet of leaf (or pinnule) organization that reveals structural fingerprints of the meristematic activities which generated it. Tracking the deployment of these activities across plant phylogeny and the fossil record reveals further evidence for the parallel evolution of leaves within Euphyllophytina. The paleontological record documents leaf evolution within several clades of Paleozoic euphyllophytes and provides direct evidence for parallel changes in pinnule structure and leaf venation in each [80]. Specifically, the fossil record demonstrates that in each of at least four clades (i.e., seed plants, ferns, equisetaleans, and progymnosperms) the most ancient representatives produced ultimate lateral units (e.g., pinnules) that had linear laminar segments with marginal vein endings, and that successively more recent representatives progressed through parallel modifications to (1) divergent venation with marginal vein endings; (2) convergent venation with marginal vein endings; (3) reticulate venation with marginal vein endings; and (4) reticulate venation with internal vein endings (summarized by Rothwell et al. [3]). Although the complete series of structural/meristematic modifications was achieved in only ferns and seed plants, these parallel evolutionary trajectories of leaf venation represent structural fingerprints for a succession of parallel changes in the meristems that contributed to the evolution of euphyllophyte leaves (or their ultimate segments in the case of compound leaves) in all four clades.

### Secondary growth

The modularity of developmental regulation takes on a much broader scope if we consider the evolution of vascular cambial growth (secondary growth) and the diversity of modes of secondary growth that have arisen among tracheophytes. One of the major unanswered questions regarding the evolution of secondary growth is whether vascular cambial growth evolved independently in different tracheophyte lineages or only once, at the base of the clade. The fossil record demonstrates that vascular cambial growth was present, outside of seed plants, in multiple currently extinct tracheophyte lineages that go back to the Middle Devonian (c. 390 million-years ago) [91]. Based on these, the traditional view has been that vascular cambial growth originated independently in the different lineages. This perspective has its roots in the perceptions that (1) the first occurrences of secondary growth in the different lineages are much younger than the origin of tracheophytes; and (2) that the anatomy of secondary tissues shows significant differences between major lineages [92].

The traditional view on the evolution of vascular cambial growth is currently reshaped by evidence coming from two directions. First, anatomical evidence suggests that some mechanisms regulating cambial growth, such as control by polar auxin transport of cambial identity and activity, are shared among major tracheophyte lineages that span the lycopsids and the euphyllophytes: lepidodendrales, equisetaleans, progymnosperms, and spermatophytes [57, 60, 93]. Second, accumulating discoveries [58, 94–96] point to much earlier origins of vascular cambial growth than previously thought, at least in the euphyllophyte clade. Together, these lines of evidence suggest that regulators of secondary growth may have become part of the euphyllophyte developmental toolkit very early in the evolution of the clade. Unfortunately, comparative genomic approaches cannot be applied to address this because, aside from seed plants, all other euphyllophyte lineages with cambial vascular growth are extinct, thus allowing recourse only to anatomy for comparative studies. Irrespective of the latter, this possibility prompts the question: could regulation of vascular cambial growth have originated in the common ancestor of euphyllophytes, or even the common ancestor of euphyllophytes and lycopsids?

**Fig. 6:**
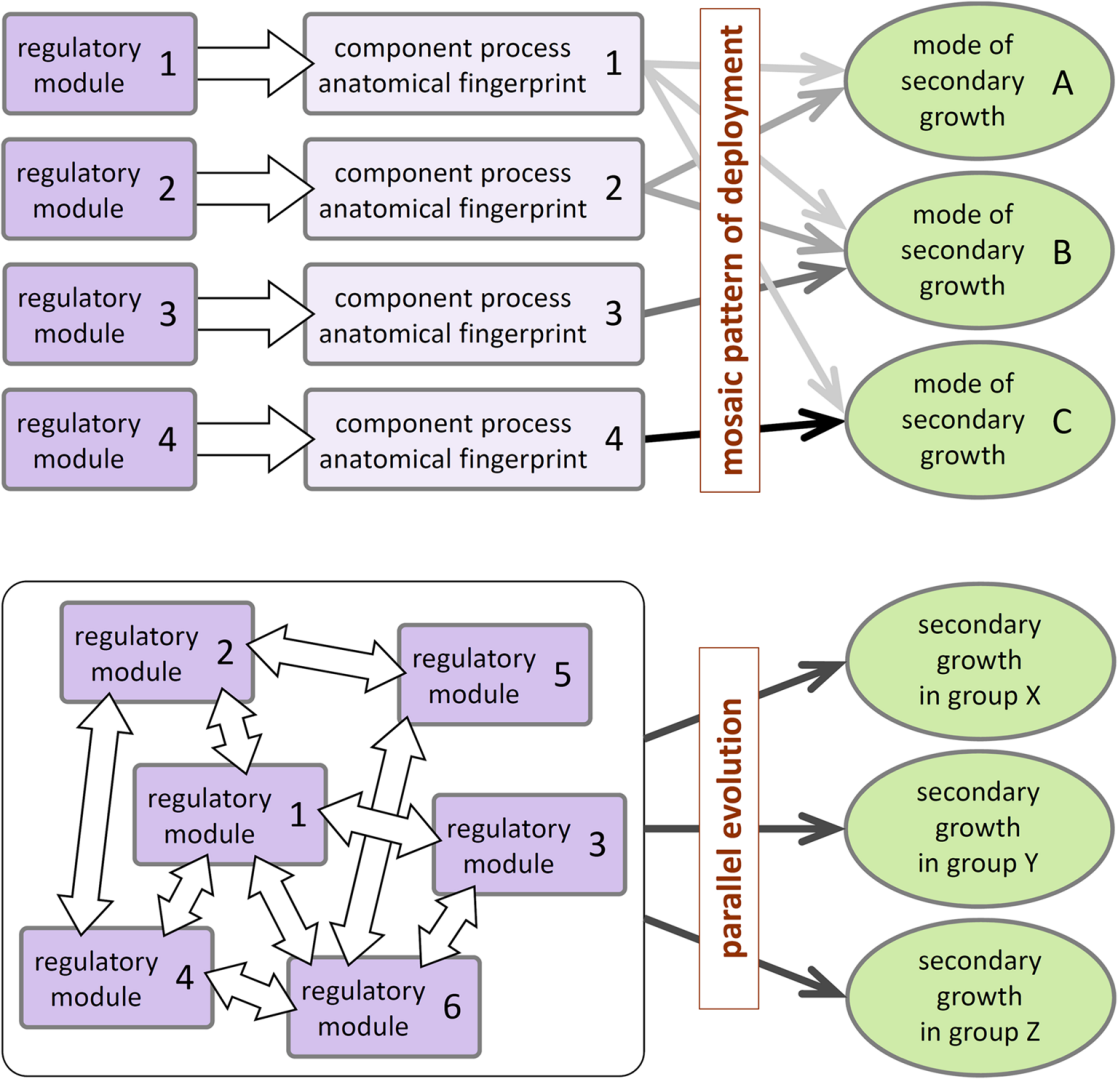
A perspective proposed by Tomescu and Groover [45] (top panel) regards vascular cambial growth as a complex modular developmental feature that is the sum of multiple component processes, each controlled by an independent regulatory module. In this perspective, component processes are deployed in a mosaic pattern among plant lineages, and their different combinations result in as many distinct modes of secondary growth. If each component process leaves a structural fingerprint in the anatomy of secondary tissues, the combinations of component processes can be inferred for the modes of secondary growth observed in the fossil record. This perspective allows for a basic set of component processes that could have defined a hypothetical single common origin of secondary growth across tracheophytes (or across euphyllophytes), underpinned by a basic toolkit of corresponding regulatory modules representing a deep homology (sensu Shubin et al. [99]) in the clade. In the traditional perspective on secondary growth (bottom panel), the implicit assumption was that of vascular cambial growth as a unitary developmental feature that was assembled de novo in each taxonomic group that evolved secondary growth independently and in parallel with other groups

**Fig. 7:**
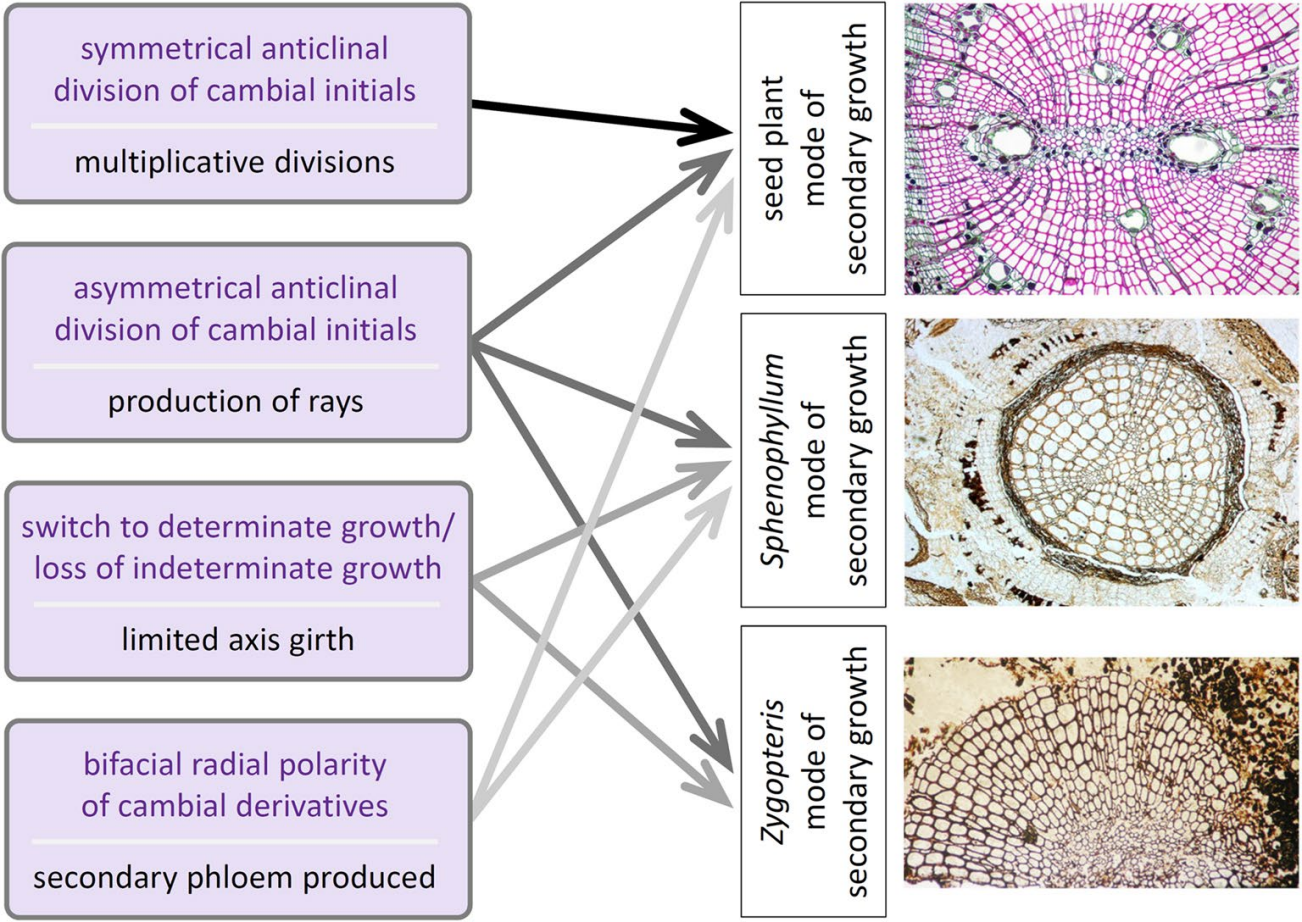
Structural (anatomical) fingerprints (in black, at left) preserved in the secondary tissues of plants living and extinct provide evidence for specific component processes of vascular cambial growth (in purple, at left) and the activity of their corresponding regulatory modules. Different combinations of such fingerprints define the distinct modes of secondary growth that differentiate seed plants from extinct sphenophyllalean sphenopsids and zygopterid ferns

To begin answering this question, Tomescu and Groover [45] have proposed an updated perspective that approaches vascular cambial growth as a complex developmental process that is highly modular (Fig. 6). In this perspective, the diverse anatomies of secondary tissues seen in different extinct lineages (and which represent diverse modes of secondary growth) reflect a mosaic pattern of expression of distinct, more-or-less independent developmental regulatory modules. Although they are as yet poorly circumscribed or simply unidentified [45], these hypothesized regulatory modules are thought to be individually responsible for different component processes that comprise secondary growth (Fig. 7)—e.g., symmetrical or asymmetrical anticlinal divisions of cambial cells, bidirectional production of new tissues. The distinctiveness and independence of the hypothesized regulatory modules are supported by anatomical observations and developmental experiments and could be tested, in principle, by altering the activity of different modules, when the regulatory interactions that control vascular cambial growth are better circumscribed.

Importantly, the activity of the different regulatory modules proposed by Tomescu and Groover [45] can be recognized based on specific anatomical fingerprints that are preserved in the wood (secondary xylem) and adjacent tissues of plants, including fossil plants. The presence or absence of these fingerprints in the wood of different lineages (Fig. 7) suggests that the regulatory modules are deployed differently among different lineages, living and extinct—some are shared among multiple lineages, while others are apomorphic for distinct lineages. Thus, information preserved in fossils and an understanding of structural fingerprints characteristic for specific developmental processes, combined in the context of an evolutionary-developmental perspective that is rooted in modularity of developmental regulatory mechanisms, can contribute to the construction of testable hypotheses about the evolutionary origins of secondary growth.

## Modularity and hierarchy

Within the paradigm of modularity in developmental regulation, information preserved in fossils and recognized as structural fingerprints for specific developmental regulators can also lead to inferences of hierarchy in the deployment of regulatory modules. An example is the case of the regulatory mechanisms that underlie the reproductive morphologies of living and extinct equisetaleans of the family Equisetaceae. The strobilus of *Equisetum* has been for a long time a puzzle in terms of homology and morphological evolution. The different types of reproductive morphologies found in fossil relatives of *Equisetum* that go back to the Permian (c. 290 million-years ago) had created a stalemate in the interpretation of the homology of the strobilus (reviewed by Ref. [47, 97]). The contradictory homology implications of the different types of reproductive morphologies stemmed from rigid application of the morphological model of the shoot as an alternation of nodes and internodes. In brief, the frustrating question was: Are the sporangium-bearing appendages (sporangiophores) attached at the nodes or along the internodes? This was important for understanding whether the strobilus of *Equisetum* is homologous to multiple nodes, each bearing a single whorl of sporangiophores, or to a single internode with multiple sporangiophore whorls attached along it. This is a fundamental question with implications for morphological evolution in one of the major tracheophyte lineages—represented today solely by the genus *Equisetum*, the equisetalean clade in an excellent example of a long phylogenetic branch wherein homology issues can only be resolved by querying the rich fossil record of the group [98].

Studies of development in living *Equisetum* show that shoots grow as a result of the combined activity of the apical meristem, which generates phytomers, and intercalary meristems, which are responsible for elongation of the internode in each phytomer. This suggested that an emphasis on the phytomeric structure of the shoot, rather than the node-internode alternation, may provide a more appropriate paradigm within which to understand homology in the *Equisetum* strobilus and, more broadly, in equisetacean reproductive morphology [47]. At the same time, current understanding of plant developmental regulation indicates (1) that meristems of all types are equivalent in their fundamental capacities, including the capacity to transition to reproductive growth (except for root apical meristems); and (2) that at least some of the regulatory mechanisms effecting this transition are shared broadly among tracheophytes [47]. Together, these observations led to the hypothesis that in equisetaceans the switch to a reproductive developmental program happens in the intercalary meristems responsible for internode elongation and, as a result, sporangiophore whorls are produced along the internodes of fertile phytomers and follow a basipetal sequence of maturation (Fig. 3j).

**Fig. 8:**
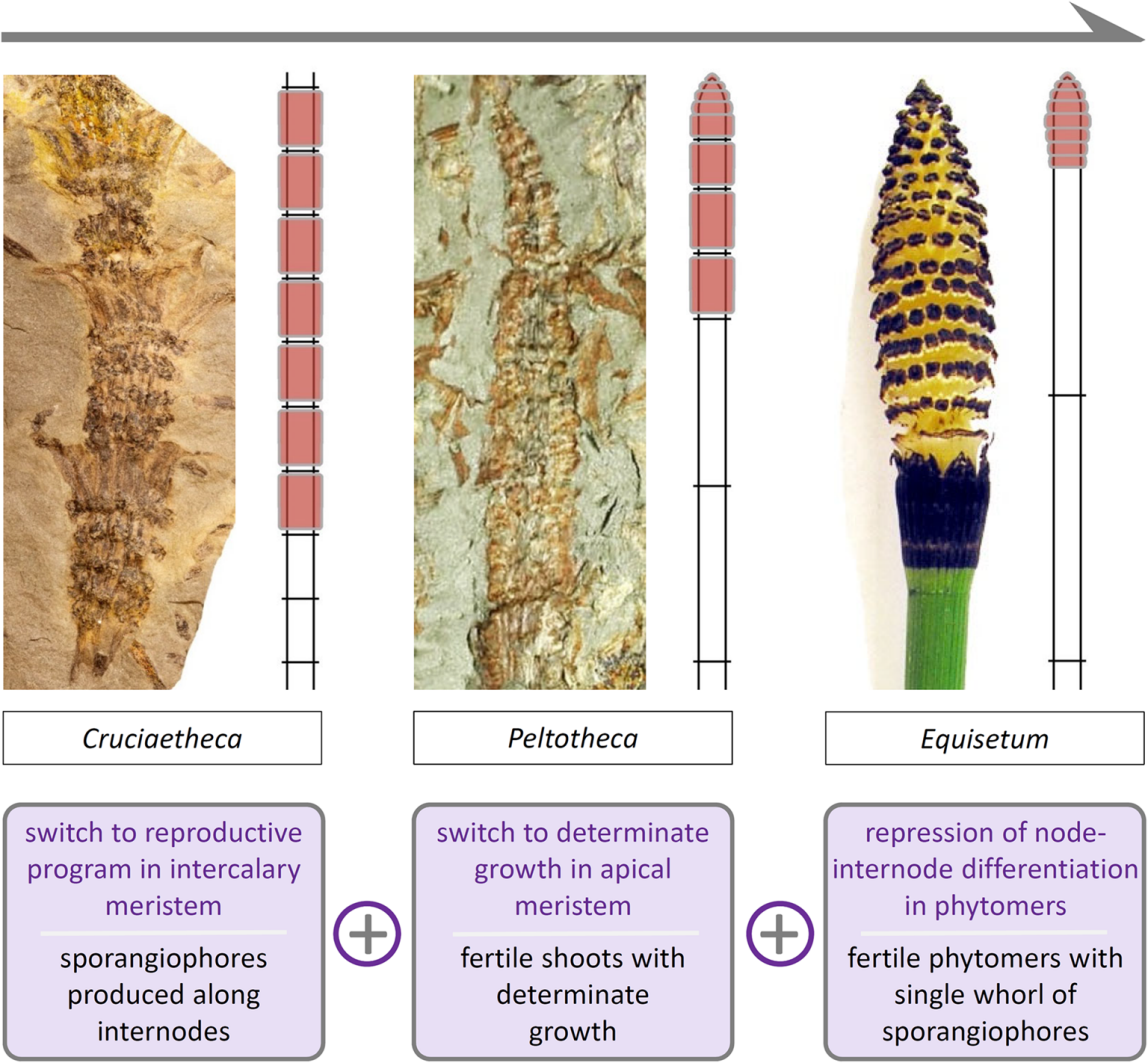
The realization that a reproductive program can be activated in the intercalary meristem of individual equisetacean internodes, leading to development of sporangiophores along them, opened up a new avenue for interpreting the reproductive structures of extinct (*Cruciaetheca*, *Peltotheca*) and living (*Equisetum*) equisetaceans as illustrating a cumulative sequence of deployment (gray arrow at top) of independent regulatory modules for three developmental processes (in purple, at bottom) responsible for the different features (in black, at bottom) that characterize specific reproductive morphologies; cross bars separate phytomers in the shoot diagrams and phytomers bearing sporangiophores are red; modified from [47]

The hypothesis of reproductive growth in internode intercalary meristems generates predictions (i.e., hypotheses) about morphological patterns produced by such a mode of development. These morphological patterns can be used as structural fingerprints (i.e., hypothesis tests), which can be recognized in the equisetacean fossil record, confirming the presence of intercalary reproductive growth (Fig. 3i, j), the only instance of its kind known in tracheophytes [47]. This confirmation provides an updated framework for understanding the origin of the *Equisetum* strobilus and of other reproductive morphologies present among equisetacean equisetaleans. These different morphologies are best explained as resulting from deployment of independent regulatory modules in a hierarchic sequence (Fig. 8): the regulatory modules (1) turn on reproductive growth in the phytomer, (2) lead to determinate apical growth, and (3) repress node-internode differentiation and intercalary meristematic activity in the fertile phytomers, respectively. Whether these hypotheses on the existence and functions of regulatory modules could be tested experimentally by altering developmental regulatory pathways (e.g., repressing growth determinacy in the strobilus meristem by overexpressing *KNOX I* family genes) will depend on our ability to genetically manipulate living *Equisetum*, a capability that has yet to be achieved.

From an epistemic standpoint, this case study demonstrates how a hypothesis generated by data from living plants is tested and confirmed using data from the fossil record [61]. In turn, this provides a framework for subsequent hypotheses that included data from living *Equisetum* and fossil plants, to offer a novel explanation, involving a hierarchy of regulatory modules, of the origin of the *Equisetum* strobilus and other reproductive morphologies of fossil equisetaleans. This updated perspective on the *Equisetum* strobilus generates further hypotheses about evolution and the deep fossil record, explaining the origin and evolution of the equisetalean sporangiophore, all of which are possible only because developmental and evolutionary data have been preserved in the fossil record.

## Conclusions

The paleontological record provides the best evidence for evolutionary pattern. Using structural fingerprints for plant development, we can also address fundamental questions about evolutionary process. Studies applying the epistemic framework of this paleo-evo-devo perspective and methodology illuminate our understanding of how evolution proceeds by successive modifications of plant development, which are controlled, in turn, by the activities of regulatory genes and growth regulators. This approach further clarifies that developmental regulation of plant growth is both modular and hierarchical. When coupled with another base of knowledge informed by the fossil record—our understanding of the overall pattern of plant phylogeny—characterization of such developmental modules, of the lineages in which they have been deployed, and of the order in which they have accumulated in divergent lineages, provide a backbone for identifying both the specific processes and the patterns by which evolution has proceeded. Continued exploration of three directions—(1) the composition, structure, and functioning of gene regulatory networks that underpin all aspects of the morphological variety seen across the diverse extant plant lineages; (2) the distinct morphological and anatomical signatures (i.e., structural fingerprints) of regulatory modules that are shared among multiple extant lineages; and (3) the occurrence of such fingerprints in the fossil record, across geologic time and phylogenetic space—will lead to deeper and more meaningful integration of data from the fossil record in the overall tapestry of the evolution of development throughout the history of plant life.

## Data Availability

Not applicable.

## Glossary

Acropetal: (1) movement (e.g., of a hormone) from the base toward the apex of an organ. (2) Pattern of tissue maturation along a structure or organ, in which the basal region is the earliest to mature and tissue maturation progresses toward the tip (distal region) of the structure or organ. Antonym: basipetal.
Anticlinal division: Orientation of the plane of cell division perpendicular to the outer surface of an organ.
Axis (pl.: axes): Ancestral vegetative organ of polysporangiophytes and tracheophytes that branches to produce more than one terminal sporangium, and that has, via evolution, given rise to stems, leaves, and roots that characterize the sporophytes of living vascular plants.
Basipetal: (1) movement (e.g., of a hormone) from the apex toward the base of an organ. (2) Pattern of tissue maturation along a structure or organ, in which the apical region is the earliest to mature and tissue maturation progresses toward the base (proximal region) of the structure or organ. Antonym: acropetal.
Derivative of an apical cell (apical cell derivative): Cell produced directly by the division of an apical cell.
Determinate growth (n., determinacy): Growth pattern in which growth ceases once a set developmental checkpoint is reached. Antonym: indeterminate growth.
Embryophytes: Major group of streptophytes that produce an embryo within the archegonium, with living representatives that are assignable to vascular plants and bryophytes (i.e., mosses, liverworts and hornworts). The term embryophytes is synonymous to land plants and Kingdom Plantae.
Equisetaleans: Major clade of vascular plants that includes living *Equisetum* and fossil representatives that extend back through time to at least the Late Devonian.
Euphyllophytes: One of the two major clades of vascular plants that includes as living representatives the flowering plants, gymnosperms, ferns (marattialeans, ophioglossaleans, and leptosporangiates), equisetaleans (i.e., *Equisetum*), and psilotaleans (i.e., *Psilotum* and *Tmesipteris*); informal name for Sub-division Euphyllophytina of Kenrick and Crane [21]. Euphyllophytes are the sister group of lycophytes (Sub-division Lycophytina). Several extinct pteridophyte-grade groups (including the earliest representatives of the clade, named trimerophytes) are also euphyllophytes.
Gametophyte: Haploid multicellular phase of the embryophyte life cycle that develops by mitosis from a spore, and that consists of a vegetative body and one or more gametangia that produce gametes.
Indeterminate growth: Growth pattern in which growth continues indefinitely throughout the life span of the organism. Antonym: determinate growth.
Intercalary meristem: The meristematic region at the base of each internode, found in some plant groups, such as the equisetaleans and grasses (Poaceae).
Internode: Length of stem between two successive positions where leaves are attached (i.e., nodes).
Lycophytes: One of the two major clades of vascular plants that includes as living representatives the lycopsids *Lycopodium* s.l., *Phylloglossum*, *Selaginella*, and *Isoetes*; informal name for Sub-division Lycophytina of Kenrick and Crane [21]. Lycophytes are the sister group of euphyllophytes (Sub-division Euphyllophytina). Several extinct groups, including lepidodendrid and pleuromeialean lycopsids, drepanophycaleans, and the earliest representatives of the clade, named zosterophylls, are also lycophytes.
Merophyte: Group of clonally related cells resulting from sequential cell divisions that originate in a single derivative of the apical cell of a meristem. The arrangement of merophytes with respect to each other may reflect the order and pattern of cell divisions by which they have been produced.
Modularity, module: Property of complex systems that refers to the relative degrees of connectivity or integration between component parts of the system. Within a modular system, a module is a unit that is tightly integrated internally (by interactions among a subset of the component parts of the system) but relatively independent from other such units.
Node: Position along a stem where one or more leaves are attached.
Phytomer: Modular unit of a shoot consisting of one node (with the attached leaf) and the subtending internode.
Polysporangiophytes: The clade of embryophytes (land plants) that share the branched sporophyte as a synapomorphy (informal name for Super-division Polysporangiomorpha of Kenrick and Crane [21]).
Psilotaleans (Psilotales): The clade of homosporous vascular plants consisting of the two living genera *Psilotum* and *Tmesipteris*. No fossils of this clade have been discovered to date.
Regulatory module: Subset of regulatory interactions (i.e., module; see definition of modularity above) that are tightly integrated internally, but can act largely independent of other such subsets (or modules) of a broader system of regulatory interactions and is responsible for a well-circumscribed developmental or morphological outcome.
Rhizomorph: Rooting organ of isoetalean lycophytes, including the living *Isoetes* and lepidodendralean trees, that is derived from (i.e., homologous to) a shoot or shoot system.
Shoot: Vegetative organ system of vascular plants that consists of a stem and the leaves that it produces.
Sporangiophore: Reproductive organ of equisetaleans consisting of an appendage bearing sporangia. Sporangiophores are thought to have evolved from fertile lateral branching systems of trimerophyte-grade euphyllophytes (see definition of euphyllophytes above). In the only living equisetalean, *Equisetum*, sporangiophores are peltate in shape, with a narrow stalk and a broad head that bears sporangia on its underside (i.e., the side that faces toward the subtending stem).
Sporophyte: Diploid phase of the embryophyte life cycle that develops by mitosis from a zygote, passes through an embryo stage, and consists of a vegetative body that bears one or more sporangia, which produce haploid spores by meiosis.
Stem: Evolutionarily derived organ of vascular plants that consists of an alternation of nodes and internodes, as well as a succession of phytomers, bears leaves at the nodes, has complex internal structure, and may have indeterminate growth (see definitions of axis, internode, node, and phytomer above).
Strobilus (pl.: strobili): Aggregation of sporangium-bearing appendages (e.g., leaves, sporangiophores) at the tip of a shoot with determinate growth.
Structural fingerprint: Morphological or anatomical feature that is the result of a developmental process underpinned by a specific regulator (set of genes/gene interactions/regulatory module), and whose presence in an organism is used as evidence for the activity of that regulator.
Thalloid: Type of plant body without complex organization, especially lacking distinct stems, leaves or roots. Many bryophytes have thalloid sporophytes, and many homosoprous vascular plants have thalloid gametophytes.
Tracheophyte: Another name for vascular plants, a group characterized by sporophytes possessing specialized water- and photosynthate-conducting tissues, which include specialized water-conducting cells (i.e., tracheids, vessel elements).
Transformational series: A sequence of different species that depict the transformation of one specific type of structure to another. Transformational series of fossils through time constitute the traditional paleontological evidence for organismal (morphological) evolution.
